# Fossil fuel methane emissions likely underestimated in a model based on atmospheric δ^13^C trends

**DOI:** 10.1073/pnas.2507837122

**Published:** 2025-06-13

**Authors:** Benjamin T. Uveges, Robert W. Howarth, Jed P. Sparks

**Affiliations:** ^a^Department of Ecology and Evolutionary Biology, Cornell University, Ithaca, NY 14853

Based on trends in δ^13^C of atmospheric methane over time, Michel et al. concluded that increased emissions from microbial sources are primarily responsible for the large growth of methane in the atmosphere since 2008 (1). They suggested these microbial emissions increased by 22 Tg/y between 2008 and 2020 and by an additional 32 Tg/y between 2020 and 2022, compared to only 3 Tg/y for fossil fuels between 2008 and 2020 and no increase in 2020 and 2022. We believe that they may have significantly underestimated the increase in emissions from fossil fuels since 2008. Here, we demonstrate our concern by detailing the rapid increase in methane emissions from just one sector of fossil fuels, natural gas in the United States, as well as illustrating how a very modest, feasible change in model input parameters can fully accommodate the observed shift.

The most striking change in fossil fuel production globally since 2008 has been the shale gas revolution in North America. Indeed, shale gas was not commercially viable until early this century, yet it now makes up more than 80% of all gas production in the United States, which is currently the largest producer and exporter of natural gas globally (2). Increased methane emissions from US natural gas production and distribution are globally significant and far exceed the changes estimated by Michel et al. for *all* fossil fuels (Table 1). We consider it unlikely that increased emissions from natural gas in the United States has been offset by decreased emissions from other fossil fuels and from natural gas elsewhere, as the consumption of all fossil fuels continued to grow globally through 2022, including for oil and coal in addition to natural gas (3).

**Table 1.** Methane emissions from natural gas production, export, and use in the United States for 2008, 2020, and 2022 (Tg/y)

	2008	2020	2022
Upstream & midstream emissions (other than for LNG exports)^*^	12.6	20.3	21.1
Downstream distribution emissions^†^	3.2	5.1	5.3
Emissions from LNG exports from the United States^‡^	0	2.2	3.5
Total natural gas emissions	15.8	27.8	29.9
Change in emissions from preceding time point	–	+12.0	+2.1

^*^Calculated from an emission rate of 2.95% of production, the mean value from a recent review based on almost one million observations (4); production data and LNG exports are from Energy Information Agency of US Dept. of Energy as recently summarized (2); LNG exports are subtracted from total production, since LNG emissions are considered separately.

^†^Calculated from an emission rate of 2.46% of gas fed through urban distribution systems, the mean value from a recent review (5); assumes 30% of natural gas used in the US is fed into downstream gas distribution systems for consumption in homes and commercial buildings (6).

^‡^LNG exports are based on data from Energy Information Agency; methane emission estimates are for average tankers as described in a recent synthesis (2).

To try and reconcile this apparent observation–model mismatch, we suggest that Michel et al. may have overestimated the amount of microbial methane emissions due to their choice of δ^13^C value for methane from microbial sources. Outputs from global box models are very sensitive to input parameters, and small changes in rates of biomass burning and the isotopic composition of shale gas can lead to major changes in conclusions (7, 8). In this case, Michel et al. assumed a microbial source δ^13^C_CH4_ value of −61.7‰ (1). If increased microbial emissions in recent years were driven primarily by changes in arctic and boreal fluxes which have δ^13^C_CH4_ values of −68.0 to −71.0‰ on average (9, 10), the choice of −61.7‰ would lead to an overestimate of the total microbial flux, with a concomitant underestimate of fossil fuel emissions. To test this hypothesis, we modified the δ^13^C_CH4_ of microbial emissions at each step-change point used by Michel et al. (2008, 2014, and 2020) and recalculated the inferred microbial and fossil fuel-derived methane fluxes. In short, we assumed that any “new” microbial emissions above the 2007 baseline had an isotopic composition comparable to arctic/boreal wetland emissions (δ^13^C_CH4_ = −68.0‰ or −71.0‰). Our recalculated changes in fossil fuel–derived methane emissions (Table 2) can more than accommodate those observed from US shale gas (2), as well as additional sources outside of the United States.

**Table 2.** Comparing fossil fuel–derived methane emission observations with estimates from different parameterizations of box model of Michel et al. (Tg/y)

	Change in fossil fuel derived methane emission rate (Tg/y)			
Year of change	Original model (1)	US shale gas estimates (2)^*^	New calculations, δ^13^C_arctic_ = −71‰^†^	New calculations, δ^13^C_arctic_ = −68‰^†^
**2008**	10	unk.	15	13.8
**2014**	3	6	10.8	9
**2020**	0	6	11.4	8.7
**2022** ^‡^	–	2.1	–	–

^*^Estimates for the change in US shale gas methane emissions were calculated for the period between 2008 and 2020 (2). Here, the 12 Tg/y change shown in Table 1 has been equally split across the 2008 to 2014 and 2014 to 2020 time periods.

^†^δ^13^C_arctic_ represents the δ^13^C_CH4_ value used for microbial methane emissions above the 2007 baseline. In practice, when using δ^13^C_arctic_ = −68‰, this resulted in microbial emission δ^13^C_CH4_ values of −61.9‰, −62.2‰, and −62.5‰ at the 2008, 2014, and 2020 step changes respectively.

^‡^The model used by Michel et al. (1) did not show a change in rates between 2020 and 2022 specifically, but rather modeled a single step change in 2020.
